# The impact of alertness vs. fatigue on interrogators in an actigraphic study of field investigations

**DOI:** 10.1038/s41598-023-32975-w

**Published:** 2023-04-15

**Authors:** Zlatan Krizan, Anthony J. Miller, Christian A. Meissner, Matthew Jones

**Affiliations:** 1 Department of Psychology, Iowa State University, Ames, IA 50010 USA; 2 Evocavi, LLC, Oro Valley, USA

**Keywords:** Psychology, Risk factors

## Abstract

Investigative interviews (e.g., interrogations) are a critical component of criminal, military, and civil investigations. However, how levels of alertness (vs. sleepiness) of the interviewer impact outcomes of actual interviews is unknown. To this end, the current study tracked daily fluctuations in alertness among professional criminal investigators to predict their daily experiences with actual field interviews. Fifty law-enforcement investigators wore a sleep-activity tracker for two weeks while keeping a daily-diary of investigative interviews conducted in the field. For each interview, the investigators indicated how well they established rapport with the subject, how much resistance they encountered, how well they maintained their own focus and composure, and the overall utility of intelligence obtained. Daily alertness was biomathematically modeled from actigraphic sleep duration and continuity estimates and used to predict interview characteristics. Investigators consistently reported more difficulties maintaining their focus and composure as well as encountering more subject resistance during interviews on days with lower alertness. Better interview outcomes were also reported on days with subjectively better sleep, while findings were generally robust to inclusion of covariates. The findings implicate adequate sleep as a modifiable fitness factor for collectors of human intelligence.

## Introduction

Collecting human intelligence is an essential activity across law-enforcement, safety, and homeland security sectors. Each day, thousands of law-enforcement officers interview suspects, victims, and witnesses, while federal investigators pursue information about critical safety incidents and intelligence officers debrief subjects about information relevant to national security^1,2^. While interrogations in criminal cases are often directed toward soliciting incriminating admissions or confessions, most investigative interviews aim to solicit a large quantity or rich information that can be verified by other intelligence or evidence, as is the case for witness interviews and homeland security investigations^2,3^.

Critically, investigative interviewers and interrogation professionals continually face challenging informational and social context requiring vigilance and complex decision making, as they face distinct types of interview subjects (e.g., debriefing a traumatized victim vs. interrogating a terrorist suspect) amid the need to continually update and reflect on case evidence^4^. This taxes interviewers’ level of alertness, vigilance, adaptability, perseverance, and self-control essential to success of investigative interviews, as emphasized in existing guides to investigative interviewing^4–6^.

## Sleep, alertness, and fatigue

Critically, human performance and decision making is very sensitive to states of fatigue, which are driven by a variety of factors, but primarily by sleep–wake mechanisms^7^. Decades of research indicate that adequate sleep is essential for maintenance of optimal physiological, cognitive, and behavioral responses, and that disruptions to the sleep–wake cycle undermine biopsychosocial functioning across a variety of contexts^8–10^. Two key sources of disruption are sleep loss and circadian misalignment. First, sleep loss refers to extended wakefulness over time, and can involve either total sleep deprivation (skipping sleep entirely) or more common sleep restriction, with insufficient sleep over days^10,11^. First, extensive evidence indicates that consequences of extended wakefulness accumulate according to the dose–response principle, and can reverse following recovery sleep^11^. These consequences appear most severe for simple sustained attention and subjective feelings of fatigue, but also present in more complex social-cognitive processing, including impaired memory and learning, as well as suboptimal processing of social information^12^. Second, circadian misalignment refers to time periods of asynchrony between the internal biological clock (producing sleep pressure during the night) and external demands which call for mental and behavioral activity during the same period^13,14^. A common case of circadian misalignment is nighttime shift work, where the need for nighttime alertness and engagement clashes with the absence of the alerting signal produced by the biological clock^15^. Note that the levels of alertness (vs. fatigue) are a dynamic combination of both the homeostatic sleep process (which ensures regular sleep) and the circadian sleep process^16^ (which prioritizes sleep to nighttime).

## Sleep and investigative interviewing

How sleep–wake disruptions may impact provision of intelligence among interview subjects (including suspects) or their willingness to confess has been a long-standing subject of debate, including moral debates about torture^17^. While the impact of sleep on interrogation subjects has drawn empirical attention^18,19^, how sleep impacts investigative interviewers and collectors of human intelligence has been relatively neglected.

Critically, sleep and circadian disruption is likely to be a pervasive force among professional investigators. First, the stressful nature of work-demands and unpredictable schedules suggest that a large proportion of interrogators may be sleep deprived or suffer from sleep-related disorders. Surveys reveal high rates of sleep disorders affecting more than 40% of police officers^20^ (insomnia, obstructive sleep apnea, excessive sleepiness). This research also revealed that officers who screened positive for sleep problem(s) reported more serious administrative errors, more uncontrolled anger toward suspects, and more frequent naps during meetings. These findings imply that sleep loss could undermine interrogation professionals’ ability to elicit information, maintain emotional composure, detect diagnostic cues to credibility, and switch between interview strategies.

Second, the 24-h nature of investigative work and criminal activity necessitates investigators to at least occasionally function during circadian misalignment, namely at night when circadian alerting is at minimum, resulting in general slowing of neurobehavioral responses and cognitive-affective dysregulation^13^. For example, surveys find that almost 20% of interrogations occur at night, further suggesting a frequent presence of misalignment between the ideal time for physiological functioning and the time of interrogations^1^. National security, intelligence, and clandestine operations may be even more likely to gather intelligence at night, after extended wakefulness, or during jet-lag while traveling to conflict zones. As a result, modeling how sleep–wake processes impact investigative interviews can reveal important insights about modifiable factors that underlie efficacy and outcomes of real-world investigations, while speaking to basic theoretical principles underlying the impact of sleep and circadian processes on consequential human behavior.

## Study objectives

The objective of this research was to evaluate the importance of sleep–wake functioning among investigative interviewers who collect human intelligence. Specifically, the present study tested how day-to-day differences in alertness (vs. sleepiness) predicted day-to-day outcomes of investigative interviews conducted in the field. To this end, law-enforcement officers who conduct interviews kept a 2-week diary of investigative interview activities while wearing sleep-activity trackers. Actigraphic information enabled estimating participants’ levels of alertness during any given “day” (active period) via biomathematical modeling, while diary reports enabled capturing outcomes of real-world investigative interviews conducted that day. In brief, the analyses examined if investigative interviews unfolded differently on days when the interviewers were more alert. Analyses also explored the underlying dimensions of sleep involved. Moreover, the study tested the relative importance of sleep–wake functioning relative to a broader indicator of global functioning, daily stress.

On a theoretical level, this investigation tested well-grounded hypotheses about inter-personal effects of sleep loss in a unique and highly-consequential setting. Systematic reviews of the literature suggest that problems with affect and emotion regulation, social disengagement, and withdrawal of effort are some of the most robust socially-relevant consequences of sleep disruption^9,21^. On the physiological level, both general reductions in central nervous system arousal^22^, as well as dysregulation within particular neural networks are responsible for these effects^23^ (e.g., the salience network, Krause et al.^23^). On the psychosocial level, impaired social communication, effort avoidance, and subjective distress drive augmented interpersonal behavior^24,25^. While the theory and evidence does not always point to clear hypotheses about outcomes for investigative interviews, it does clearly suggest that sleep-deprived investigators should experience affective distress, anger, and struggle with emotion control^9,21^. Less clearly, it also suggests sleepy interviewers may experience more difficulties with establishing rapport with the interview subject, due to presumed difficulties in maintaining social attention and communication^26,27^. To examine these hypotheses as well as explore potentially consequences of fatigue more broadly, the current study tracked field interviews and examined the impact of sleep fluctuations on separate investigator reports about (1) established rapport with the interview subject, (2) that subject’s apparent resistance, (3) investigator’s own composure, and (4) utility of information obtained across field interviews. Note these are considered key components of contemporary models elucidating investigative interviewing^28^.

On a practical level, often-serious consequences of fatigue and sleep loss for law enforcement, military, and intelligence collectors suggest a need for the systematic assessment of alertness in investigators, especially associations between these characteristics and operational performance. There is also a lack of objective measurement of sleep and alertness (alongside performance) in the field, with virtually all current evidence limited to one-time survey reports. Given more precise evidence, interventions and training efforts can be implemented to improve collector alertness, as has proven successful in other highly skilled, high-stakes professions (e.g., airline pilots^28^. To our knowledge, this is the first unclassified study to evaluate sleep–wake functioning as a factor in field collection of human intelligence.

## Method

### Sample and timeline

As individuals of interests were professional investigators (i.e., those who routinely conduct investigative interviews), existing professional contacts helped identify potential participants. Those who expressed interest received an enrollment packet that contained further instructions and a sleep-activity tracker. All participating officers worked as a part of a non-federal investigative or law-enforcement organization. The sample was drawn from seven institutions across Arizona, Iowa, Kansas, and Nevada. Overall, 79 officers enrolled in the study across 2019–2020 and provided at least some data, while 50 officers provided at least one day of joint actigraphic and investigative-diary data necessary for the analyses of interest. These key analyses thus involved 204 days across 50 officers, while analysis of actigraphic sleep–wake variables only (a larger set) involved 1442 days.

Out of the whole sample of 79 officers, 59 (74.7%) of the officers identified as male, and 20 (25.3%) as female. They ranged in age from 27 to 60 (mean = 42.06, SD = 8.06). Of 54 who did not decline to respond to the question on ethnicity, 95.8% identified as white and 4.2% as Hispanic. Twenty-two reported that they served in a detective role while three reported serving in a leadership role. All participants had investigative duties and conducted investigative interviews on a routine basis. Most respondents (79.6%) reported working typical morning to afternoon shifts during the study period, while 10.2% reported working from evening to late night, 8.2% working from late night to early morning, and 2% were scheduled to have multiple shifts during the study period.

First, participants received an envelope with instructions to enroll in the study, as well as a Fatigue Science Readiband^®^ actigraph. Once they contacted the laboratory and registered the Readiband with the Fatigue Science platform, they completed an online background survey that inquired about demographics and sleep health (not discussed in detail within this report). Officers indicated their preferred time for daily surveys (to accommodate varying shifts), which were then e-mailed to them across 14 days (while they wore actigraphs). Following the two-week period, officers were debriefed over e-mail and their participation and data collection were terminated.

All research procedures were approved both by the Iowa State University Office for Responsible Research and the Federal Bureau of Investigation Internal Review Board. All methods were performed in accordance with relevant guidelines and regulations. Informed Consent was obtained from all subjects. The names of participating institutions and individual-level data are not disclosed due to confidentially assurances to participants during informed consent. Otherwise, all materials, analyses scripts, and results presented in this manuscript are available on Open Science Framework (https://osf.io/69nqt/).

### Measures

Upon enrollment, participants completed a background survey which included measures of habitual sleep (see all materials on Open Science Framework). The survey also queried participants about overall well-being, exposure to traumatic events, well-being, and personality traits. Analyses of these individual differences are beyond the scope of the present report given the focus on daily fluctuations in alertness.

#### Actigraphy and alertness estimation

The Readiband^®^ sleep-activity tracker uses three-dimensional accelerometer technology (sampled at 16 Hz) to measure movement and infer sleep–wake states. It relies on an automated proprietary algorithm to determine sleep-onset time In terms of sleep–wake variables, the Readiband outputs standard characteristics, including period-specific (e.g., nightly) sleep duration, sleep-onset latency, sleep-efficiency, and Wake-After-Sleep-Onset. The inter-device reliability of the Readiband in determining sleep–wake states is very high, estimated at 95% among healthy individuals in a recent study^29^. In terms of validity, Readiband is similar to other research-grade actigraphs when evaluated relative to polysomnography^30,31^.

Critically, the device and accompanying software utilize biomathematical modeling to continually estimate real-time fatigue. Specifically, the Readiband uses the extensively validated Sleep, Activity, Fatigue, Task, and Effectiveness Model (SAFTE) developed by the U.S. Army to estimate fatigue for each 30 s period given a minimum of three days of continuous data^10,32^. To do so, this model integrates actigraphically-recorded sleep duration (time spent asleep), sleep continuity (number and duration of interruptions), alongside time of day (circadian misalignment), and sleep consistency (regularity over time). Ultimately, the algorithm yields scores representing levels of alertness (vs. sleepiness or fatigue) between 0 and 100 for each 30 s epoch (“SAFTE Scores”). These scores indicate the person’s level of alertness (vs. sleepiness) relative to their own baseline and is only generated after 72 h of continuous recording.

More specifically, the SAFTE scores track the *percentage* of the person’s optimal *baseline response speed*, based on the observed level of sleep and circadian disruption. For example, a SAFTE score of 90 indicates that a person is around 11% slower than when at their normal. As a result, SAFTE scores can also be expressed as Blood Alcohol Content that would produce a similar level of impairment in response speed (https://www.fatiguescience.com/sleep-science-technology/). The SAFTE model has been extensively validated in laboratory contexts that assess reaction time across multiple days, as well as within real-world contexts involving railroad, aviation, and military operations that measure performance and accident risks^33^. Scores around 85 or higher are considered ideal, while scores below 80 indicate around 25% slower responses, and scores below 70 indicate dangerous fatigue impairment. For example, scores below 80 indicate *threefold* increase in likelihood of attentional lapses and approximate the effects of 0.05 Blood alcohol content^34^.

#### Daily survey

Each day during a time period they marked in the background survey, participants received a text-message with a link to an online *daily* survey. In this survey, officers first reported their subjective sleep *quality* of the prior rest period (“How well did you sleep last night?”). To capture global daily functioning, they also indicated that day’s stress (“How stressed do you feel today?”), as well as the amount of time spent on self-care (“How many hours did you spend on self-care today (exercising, relaxing, hobbies)?”) on 5-point scales. They also responded to “How many servings of Caffeine have you consumed since you have woken up?”. Finally, they indicated their working hours on that day, including any court time (“Please indicate the exact hours you have worked [including court] since the last time you completed this survey?”) these were used to accurately specify rest and active periods for data analysis that reflect varying shifts.

During each daily survey, participants were asked if they conducted an investigative interview, defined as a *10-min or longer conversation aimed at obtaining specific information*. If so, they responded to the following questions regarding the interview (if a participant indicated multiple interviews that work period, they answered the same questions for each interview reported, up to 8). First, they indicated the general *time* of the interview (in three hour blocks starting at midnight and spanning twenty-four hours), the *location* of the interview (interrogation room, residence, vehicle, or other (e.g., street), and the *duration* of the interview (less than 30 min, 30–60 min, or 1 h or longer). Descriptive information for all the interviews appears in Supplemental Materials.

Critically, the investigators reported their assessment of each interview regarding their relationship with the subject (rapport and resistance), their own reactions (difficulties with focus and emotional composure), and the perceived usefulness of obtained information. First, *established rapport* was assessed by asking officers “How well was the rapport and co-operation established? (Please indicate the extent of the rapport, co-operation, and mutual respect you established with the interview subject). Second, *subject resistance* was assessed by “How difficult was it to obtain information?” (Please indicate how difficult was to obtain disclosure of desired information due to resistance from the subject?). Third, *investigator composure* was assessed with “How difficult was it to maintain your focus and emotional composure” (please indicate how difficult was it to sustain attention and control one’s emotional reactions). Fourth and final, the perceived *information utility* of obtained intelligence was assessed by “How useful was the information obtained?” (Please indicate the quantity and quality of information obtained during the interview). Responses to all these questions were made on “Not at All” (1) to “Extremely” (5) Likert-type scales.

### Variables and analyses

First, in order to estimate *average daily alertness* during work periods when officers conducted interviews, each participant’s actogram (record of sleep–wake activity from the Readiband) was examined alongside reports of working hours to determine an active work period (time span during which officers conducted interviews and were awake, even if nighttime) and a rest period (time span during which officers slept and did not work, even if daytime). Cross-referencing ensured that dates of actigraphic rest-activity periods are appropriately paired with next-day diary reports. Then, the Readiband SAFTE alertness scores from each scored epoch across these active periods were aggregated to estimate average alertness for that officer during the respective work period (“day”). Note that SAFTE scores were generated only after 72 h of continuous recording, which results in a restricted set of days for these analyses (relative to other sleep variables which are generated every recorded rest period). Furthermore, all sleep periods are factored into the SAFTE model’s sleep algorithm and have a corresponding effect on individuals’ estimates. However, naps of 30 min or less may not be recorded by the Readiband so would not be reflected by the sleep duration estimates.

Second, in order to estimate *daily interview*-*outcomes*, we aggregated ratings for each dimension across all interviews reported that day. For example, if officers reported multiple interviews on a given day (29%), their responses about established rapport were averaged across all interviews to reflect overall rapport established across interviews conducted that day (commensurate with sleep–wake variables).

While SAFTE scores were available on a moment-to-moment basis, interview experiences were recalled only once daily without exact times, precluding a finer-grained analysis. As a result, for key analyses sleep characteristics (i.e., duration, continuity) of the prior rest episode (“prior night”) were utilized as predictors of waking function reported for the subsequent active period (e.g., interview outcomes the following day).

#### Statistical precision

For inferential tests of the predictive strengths of sleep and alertness for daily interview outcomes while taking account data-clustering within individuals and day-level co-variates, we estimated fixed coefficients within multi-level models with days (Level I) nested within participants (Level II). Models were implemented in R-Studio version 2021.09.1+372 using *lmer* package, with outputs are available on OSF.

Given high variability in the number of days with interviews across investigators (with multiple investigators reporting only one or two days with interviews), the analysis focused on the day-level associations across the whole sample. To this end, scores were *grand*-mean centered, such that daily alertness, sleep, and interview variables reflected deviations from the average day in the sample, regardless of the investigator. The estimated parameters thus represent day-level linear regression coefficients between sleep and alertness on one hand, and interview outcomes on the other, accounting for clustering within participants (partial in presence of day-level covariates).

According to simulations reported by Arend Schafer, exceeding 5 day-level observations and 50 person-level observations affords at least 80% power to detect small-to-moderate Level 1 direct effects^35^. To this end, the recruitment goal was 200 day-level observations or more (regardless of nesting), also approximating similar power to detect small-to-moderate correlations. Note that large day-level variance components are expected in sleep-tracking studies^21^, which contributes to power for identifying Level I effects even with few observations per Level I unit (i.e., number of individuals^35^).

#### Distributions and outliers

Distributions of key interview and sleep variables were inspected prior to the analyses to identify potential outliers (i.e., observations 3 or more standard deviations from the mean) and to identify anomalous distributions. For nearly all reported interview outcomes, ratings spanned the entire scale range (with the exception of ‘extremely’ ratings for investigator having composure difficulties). They were also normally distributed with minor skew. Only one daily interview data point was more than 3 SD below the mean (a rating of ‘not at all’ for establishing rapport). Given that the next-higher ratings regarding rapports were common and the distribution was continuous, we retained this data point. Inspection of alertness and sleep variables (duration, wake-after-sleep-onset, subjective quality) did not reveal any outliers (results appear in the Online Supplement).

## Results

### Data coverage

The key analyses involved day-level associations between biomathematical estimates of alertness (SAFTE scores) and field interview reports. As a result, for cases of multiple interviews reported that day (29% of days), the daily averages of interview experiences were utilized as criteria, regressed on characteristics of prior sleep episodes (or that day’s SAFTE scores). Descriptive analyses of interviews themselves, however, were conducted across all individual interviews to avoid information loss. Similarly, note that descriptive analyses of sleep variables involve the largest number of data points, as interviews and diary self-reports only occurred on some days participants wore the actigraph. Analyses involving SAFTE scores also rely on a smaller sample of days than analyses with other sleep variables, given at least three days of continuous monitoring were required for estimation of SAFTE scores.

Overall, 204 days across fifty officers included information on both key variables (sleep and interviewing). On days when participants attempted diary entries at all there was very little missing data (0.41% missingness). The Readiband was worn in 82.7% of cases during the preceding rest period (i.e., 24 h) when investigative diary was available for key analyses.

### Investigative interview characteristics

Overall, the interviews reported were diverse and varied in setting, length, and occurred across the entire 24-h cycle. In general, they unfolded during the day (when most participants worked), not in controlled environments, and were relatively brief (Supplemental Materials). While most interviews occurred during typical working hours, around 25% of interviews occurred at nighttime when the circadian rhythm impairs function, namely between 9 pm and 9am. A minority of the interviews occurred in the interrogation room, patrol vehicle, or subjects’ residence (about a third), with most interviews in other locations (e.g., street). Finally, most interviews were brief, with less than a quarter of interviews longer than 30 min. Overall, the interviews and interrogations reported by the officers reflect the routine nature and the large number of brief interviews conducted during the course of everyday inquires (e.g., minor crimes) with in-house lengthy interviews typical to major crimes (e.g., homicide) occurring infrequently (see Online Supplement). Descriptive information about interview outcomes appears in Table 1.

**Table 1:** Descriptive statistics and inter-correlations of investigative interview reports.

	1	2	3	4	*M*	*SD*
1. “How well was the rapport and co-operation established?” (Rapport)		− 0.58**	− 0.31**	0.56**	3.89	1.02
2. “How difficult was it to obtain information?” (Resistance)			0.39**	− 0.52**	2.16	1.16
3. “How difficult was it to maintain focus and emotional composure” (Difficulty)				− 0.26**	1.78	1.00
4. “How useful was the information obtained?” (Utility)					3.42	1.14

Spearman non-parametric correlations across individual investigative interviews (N = 293). *p < 0.05; ** p < 0.01.

### Sleep and daytime function

Descriptive statistics for basic sleep parameters and alertness for all officers appear in Table 2. When contrasted with public health recommendation, the data reveal that the officers did not sleep for the recommended duration on a typical day (i.e., rest period), namely around 6.7 h, alongside large nightly fluctuations.

**Table 2:** Descriptive statistics and correlations of daily alertness, sleep, and well-being variables.

	1	2	3	4	5	6	*M*	*SD*
Actigraphy (Readiband)								
1. Alertness (SAFTE)		0.87**	− 0.65**	0.39**	− 0.15	− 0.1	87.21	6.89
2. Sleep duration	0.38**		− 0.51**	0.28*	− 0.04	− 0.09	403.94	47.17
3. Wake-after-sleep-Onset	− 0.28**	− 0.021		0.01	− 0.43**	0.04	39.51	25.79
Self-reports (daily diary)								
4. Sleep quality	0.16**	0.34**	− 0.17**		− 0.43**	0.17	3.2	0.61
5. Daily stress	− 0.08*	− 0.13**	0.01	− 0.39**		− 0.27*	2.27	0.66
6. Daily self-care	− 0.04	0.04	0.06	0.21**	− 0.29**		1.63	1.14

Spearman’s non-parameteric correlations across days. Day-level correlations appear *below* the diagonal (*N*_*days*_ = 168–204), while person-level correlations appear *above* the diagonal (*N*_*persons*_ = 50). *p < 0.05; **p < 0.01.

In terms of sleep-continuity, on a typical night the participants were awake nearly 40 min in bed (public health recommendations call for less than 30 min), although these estimates varied widely. Note that the shift-work often results in fragmented sleep without stable active and rest periods, which contributed to poor sleep continuity among some officers.

When inspecting estimated levels of alertness, officers were relatively alert on most days, with 87.2 SAFTE score on the average day. Nevertheless, 15% of days involved officers with suboptimal average alertness (25% slower reactions, SAFTE less than 80), and some days involved levels of impairment equivalent to being legally intoxicated in most U.S. states (i.e., BAC > 0.08). As would be expected, officers who worked the late night to the early morning shift (45 days across 4 officers) showed the lowest daily level of alertness, namely 78%. These data suggests that on many days investigators (despite relatively stable sleep–wake schedule) experienced suboptimal (occasionally dangerous) levels of sleepiness and fatigue.

### Alertness and interview outcomes

Correlations between daily sleep–wake variables and interview outcomes are presented in Table 3. Overall, they suggest trends for interview outcomes to be better on days with less fatigue, especially for encountering less subject resistance and maintaining focus and composure. Subjective sleep quality exhibited the strongest associations with interview outcomes.

**Table 3:** Associations between daily interview outcomes and daily functioning (N = 168–204 days).

	Sleep–wake				General functioning	
	Alertness (SAFTE)	Sleep duration	Wake-after-sleep-onset	Sleep quality	Daily stress	Self-care
Established rapport	0.11	− 0.11	0.04	0.11	− 0.03	0.14*
Subject resistance	− 0.18*	0.04	− 0.04	− 0.19**	0.17*	− 0.15*
Investigator composure	− 0.18*	− 0.11	0.00	− 0.34**	0.41**	− 0.10
Information utility	0.07	− 0.12	0.04	0.13	0.06	0.14*

Pearson’s correlations across investigator-days. *p < 0.05; **p < 0.01.

The inferential results from multi-level model tests with sleep–wake variables as predictors of interview outcomes (Level I) appear at the top of Table 4. Broadly, they indicate that how investigators slept before their shift and how subsequently alert they were predicted outcomes of their field interviewing. Specifically, on days investigators were more alert and slept longer, they consistently reported fewer difficulties maintaining their focus and composure during interviews, as well as encountering less subject resistance. Actigraphic sleep variables or SAFTE scores did not significantly predict other interview outcomes, although all coefficients were in the anticipated direction. Further, officers’ subjective *sleep quality* the night before predicted less resistance and better composure, as well as higher information utility obtained from the interviews. Inspection of specific sleep variables revealed weaker patterns, but implicated sleep *duration* as the key component (with it predicting better composure and rapport).

**Table 4:** Estimates of Fixed Effects from Individual Multi-Level Models of Daytime Functioning Variables as Day-Level Predictors of Investigative Interview Characteristics (N = 168–204 days).

	Sleep–wake				General l	
	Alertness (SAFTE)	Sleep duration	Wake-after-sleep-onset	Sleep quality	Daily stress	Self-care
Established rapport	0.013	− 0.002*	0.001	0.06	− 0.012	0.064
Subject resistance	− 0.023**	0.001	− 0.001	− 0.142*	0.152^◊^	− 0.098*
Investigator composure	− 0.022**	− 0.001^◊^	− 0.001	− 0.261**	0.349**	− 0.058
Information utility	0.009	− 0.001	0.001	0.12*	0.064	0.085^◊^
Regression coefficients after adjusting for daily stress (Level I), age, and sex (Level II)						
Established rapport	0.020*	0.004	0.002	0.263		0.122*
Subject resistance	− 0.022*	− 0.001	0.012	− 0.172*		− 0.601*
Investigator composure	− 0.015	− 0.001	0.011	− 0.214**		− 0.178
Information utility	0.006	− 0.001	− 0.013	0.148*		0.338

All continuous variables were grand-mean centered. ^◊^ *P ≈ *0.05; * P < 0.05; ** p < 0.*01.*

Higher daily stress predicted reports of higher subject resistance and more composure difficulties, while greater time spent on self-care among interrogators results in them indicating subjects they interviewed exhibited less resistance and provided somewhat better information. In order to evaluate the importance of sleep–wake variables for field interviews in light of global functioning and demographic differences, we further evaluated sleep–wake variables as competing predictors alongside daily stress (Level I), age, and sex (Level II, see bottom of Table 4). These results echo main analyses, as alertness remained a significant predictor of less subject resistance and also better rapport, although the coefficient for investigator composure was smaller and no longer significant. Overall, these statistical adjustments slightly reduced the size of coefficients, but did not alter the broader pattern. Subjective sleep quality remained a significant predictor of all outcomes besides rapport.

### Magnitude of associations

**Figure 1:**
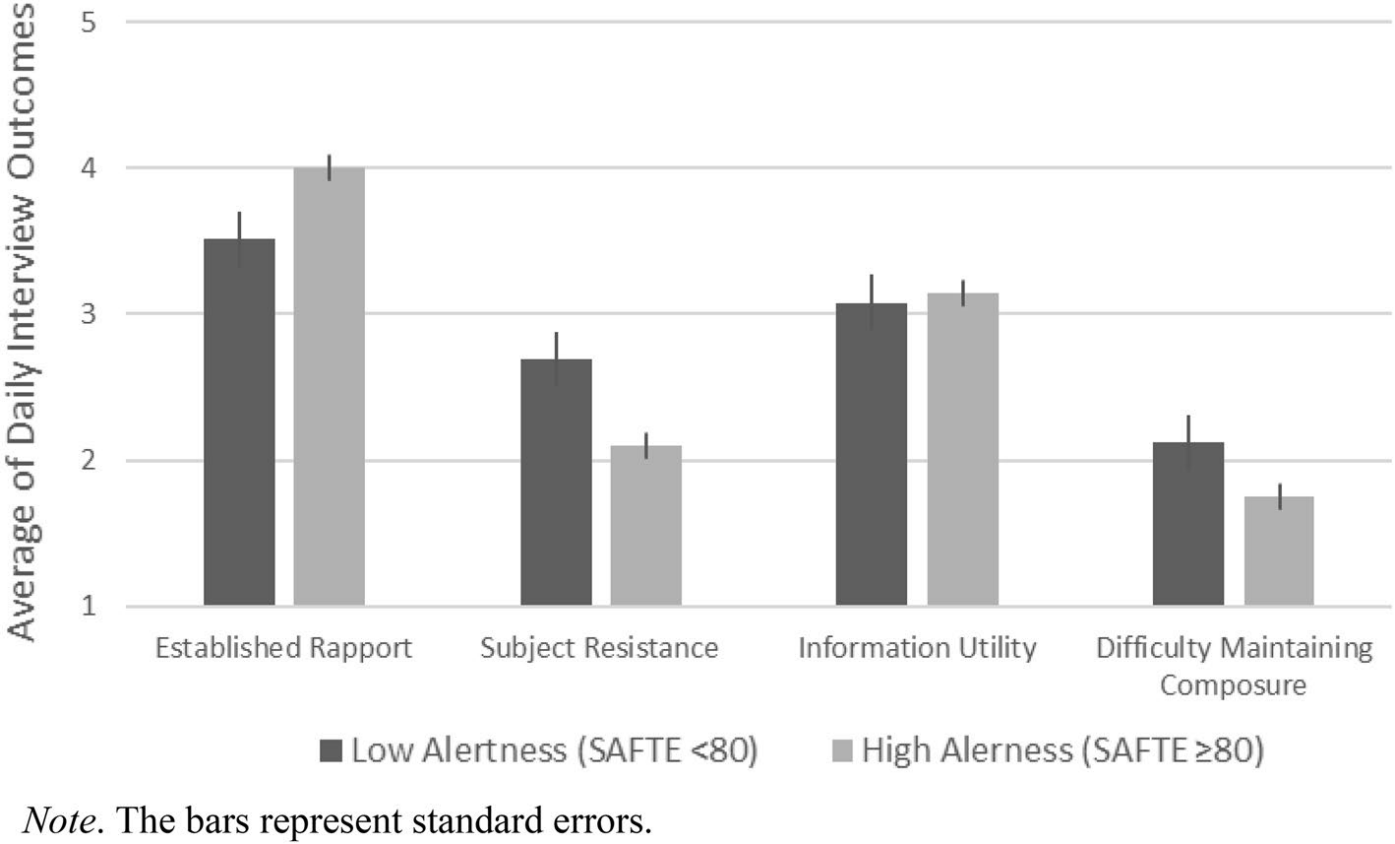
Mean differences in interview outcomes as a function of daily alertness level.

To more concretely evaluate the differences in outcomes of investigative interviews, we present the findings by separately plotting interview outcomes on days with impaired alertness (approximating a BAC ≥ 0.05 and three-fold higher likelihood of attentional lapses; http://www.fatiguescience.com/wp-content/uploads/2016/09/SAFTE-Validation-US-DoT-Railroad.pdf) from interview outcomes on days with optimal alertness (SAFTE over 80, Fig. 1). Across the board, officers tended to report poorer interview outcomes on such days (between a half and whole scale point, on average). While it is difficult to quantify the level of practical impact in this setting, the observed associations were moderate to strong by statistical standards^36^.

## Discussion

This study utilized bio-mathematical modeling of alertness (vs. fatigue) among law-enforcement interviewers to examine its impact on real-world interrogation outcomes. First, the findings confirmed expected sleep disruption among the involved officers. Although most experienced regular schedules, they often slept less than the recommended amount, had multiple nighttime awakenings, and many days with suboptimal levels of alertness. These findings dovetail prior analysis of fatigue among law-enforcement^20,37^.

Critically, the findings revealed substantive associations between levels of alertness vs. fatigue (inferred from sleep–wake behavior), subjective sleep quality, and investigative interview outcomes. The most striking findings were evident for more fatigued investigators reporting higher resistance among subjects they interviewed; this was a robust effect that changed only slightly after accounting for demographic and daily covariates. While it is possible that more fatigued days coincided with more resistant interviewees, it is also plausible that tired investigators lose patience and experience securing co-operation as especially effortful, leading to them perceiving their interview subjects as more resistant^20,21^.

Higher alertness was also associated with better investigator composure and better established rapport, although these effects were somewhat smaller and yielded less consistent effects across analyses. These associations again dovetail extensive evidence that sleep loss and fatigue impairs emotion regulation and anger control^21,38,39^. As a result, an events perceived as provoking by the interviewer may have an especially large effect on a fatigued interrogator, ultimately undermining establishing co-operation and maintaining control. While *subjective* sleep quality was the strongest predictor of interview outcomes across analyses, the common method variance (with both self-reported) likely inflated associations. Critically, the ability of actigraphically-assessed sleep among investigators to predict outcomes of investigative interviews with practically random interview subjects speaks to the importance of sleep–wake functioning in this context. For example, the bio-mathematically estimated alertness showed associations with interview outcomes often similar or larger than self-reported daily stress, despite the fact the latter shares method variance with self-reported interview outcomes.

An important limitation of this work is that interview outcomes were self-reported by the investigators, which means it is possible that fatigue shaped investigator’s *perceptions* of interviews rather than their actual behavior. While this cannot be ruled out, even in that case how investigators appraise the results of their interviews is determinative of real-world legal outcomes. In addition, this sample also had a relatively small number of night-shift officers, or interrogations that occur at night or during jet-lag (more common among the homeland security interrogators). Finally, sleep may have been underestimated due to missed naps, although that would imply conservative estimates regarding the impact of sleep on next-day interviews as analyzed here. Also, in some cases sleep-fatigue data was not available due to recording requirements, which may have impacted estimates.

Practically, most of the interviews in this sample were relatively brief (30 min or less), and thus may not represent more pronounced effects of interviewer fatigue when conducting lengthy interviews. In this vein, the impact of sleep and alertness may be even more profound in other settings. Future research should examine more objective interrogation outcomes, focus on night-time interviews and functioning during jet-lag, and consider realistic policies that may mitigate the impact of fatigue on efficacy of investigative interviewing.

## Supplementary Information

Supplementary Information.

## Data Availability

Due to confidentiality assurances to participants, individual-level data cannot be publicly shared. All procedures, materials, analyses, and summary results are available on the Open Science Framework (https://osf.io/69nqt/). For further inquiris contact Zlatan Krizan (zkrizan@iastate.edu).
