# Supplementary material for: The impact of alertness vs. fatigue on interrogators in an actigraphic study of field investigations
Source: Sci Rep. 2023 Apr 15;13:6135. doi: 10.1038/s41598-023-32975-w (PMC10105754; doi:10.1038/s41598-023-32975-w)
Supplement: Supplementary file 1 — Supplementary Information. [file 41598_2023_32975_MOESM1_ESM.docx]

**Supplemental Materials**

Data Overview

- Total officers enrolled who provided *both* actigraphy & diary data
  - 79
- Total days with *incomplete* actigraphy
  - 1784
- Total days with *complete* actigraphy
  - 1442
- Total number of interviews
  - 296
- Total days with *both* interviews & actigraphy
  - 204
- For 91.3% of the total cases no interview data was provided

Supplemental Table 1: Interview Descriptive Statistics

| Interview Descriptives | Min | Max | Mean | SD |
| --- | --- | --- | --- | --- |
| Established Rapport | 1 | 5 | 3.89 | 1.02 |
| Subject Resistance | 1 | 5 | 2.16 | 1.16 |
| Information Utility | 1 | 5 | 3.42 | 1.14 |
| Investigator Composure | 1 | 5 | 1.78 | 1.00 |

| Interview Locations | | Percent |
| --- | --- | --- |
|  | Interrogation Room | 25.7 |
|  | Patrol Vehicle | 6.2 |
|  | Subject Residence | 11.0 |
|  | Other | 57.1 |

| Interview Length | | Percent |
| --- | --- | --- |
|  | 10-30 minutes | 73.5 |
|  | 30-60 minutes | 21.6 |
|  | More than 60 minutes | 4.8 |

Supplemental Table 2: Interview Characteristics

| **Interviews on a given day** | | | | | |
| --- | --- | --- | --- | --- | --- |
|  | | Frequency | Percent | Valid Percent | Cumulative Percent |
|  | .00 | 2130 | 91.3 | 91.3 | 91.3 |
|  | 1.00 | 144 | 6.2 | 6.2 | 97.4 |
|  | 2.00 | 43 | 1.8 | 1.8 | 99.3 |
|  | 3.00 | 10 | .4 | .4 | 99.7 |
|  | 4.00 | 3 | .1 | .1 | 99.8 |
|  | 5.00 | 3 | .1 | .1 | 100.0 |
|  | 9.00 | 1 | .0 | .0 | 100.0 |
|  | Total | 2334 | 100.0 | 100.0 |  |

- The number of *days* in which at least one interview was conducted is 204.

| Interviews | | |
| --- | --- | --- |
| N |  | 204 |
| Mean | | 1.45 |
| Median | | 1.00 |
| Mode | | 1.00 |
| Sum | | 296.00 |

- On 70.6% of days with an interview, there was only one interview conducted.

| **Interviews Counts** | | | | | |
| --- | --- | --- | --- | --- | --- |
|  | | Frequency | Percent | Cumulative Percent |  |
|  | 1.00 | 14 | 6.2 | 70.6 |  |
|  | 2.00 | 43 | 1.8 | 91.7 |  |
|  | 3.00 | 10 | .4 | 96.6 |  |
|  | 4.00 | 3 | .1 | 98.0 |  |
|  | 5.00 | 3 | .1 | 99.5 |  |
|  | 9.00 | 1 | .0 | 100.0 |  |

Supplemental Table 3: Officer Characteristics

- 52 officers identified as white, 2 as Hispanic, and 25 provided no answer.

| **Officer Ethnicity** | | | | | | | |
| --- | --- | --- | --- | --- | --- | --- | --- |
|  | | White | Black | Hispanic | Asian | Native American | No Answer |
|  |  | 52 | 0 | 2 | 0 | 0 | 25 |

- There were 20 female and 59 male officers

| **Officer Gender** | | | | | |
| --- | --- | --- | --- | --- | --- |
|  | | Frequency | Percent | Cumulative Percent |  |
|  | Female | 20 | 25.3 | 25.3 |  |
|  | Male | 59 | 74.7 | 100.0 |  |

- Physical characteristics of officers showed a mean BMI of 30.9 and average age of 42.47.

| **Physical Characteristics** | | | | |
| --- | --- | --- | --- | --- |
|  | Age | Weight | Height | BMI |
| Mean | 42.47 | 212.93 | 69.53 | 30.90 |
| Median | 44 | 209.94 | 70.00 | 29.98 |
| SD | 7.68 | 48.19 | 4.17 | 6.30 |
| Range | 27-60 | 127-345 | 54-78 | 21.17-30.75 |
|  |  |  |  |  |

Supplemental Table 4: Sleep Variables

| Officer Averages | | | | | |
| --- | --- | --- | --- | --- | --- |
|  | Minimum | Maximum | Mean | SD |  |
| Actigraphy Variables |  |  |  |  |  |
| Alertness | 61.34 | 96.26 | 87.21 | 6.89 |  |
| Minutes in bed | 319.83 | 588.00 | 481.39 | 46.32 |  |
| Minutes asleep | 276.61 | 505.00 | 403.94 | 47.17 |  |
| WASO | 8.89 | 111.07 | 39.51 | 25.79 |  |
| Wake episodes | .86 | 9.00 | 3.32 | 1.71 |  |
| Awakenings per hour | .11 | 1.28 | .52 | .30 |  |
| Efficiency | 69.12 | 94.58 | 84.52 | 6.11 |  |
| Onset variance | -91.19 | 65.03 | -1.81 | 17.59 |  |
| Wake variance | -58.50 | 87.92 | .86 | 14.27 |  |
| Hours asleep past week | 31.98 | 59.59 | 47.54 | 5.04 |  |
| Daily Diary Variables |  |  |  |  |  |
| Sleep Quality | 2.75 | 9.24 | 6.72 | 1.76 |  |
| Self Care | .00 | 5.13 | 1.63 | 1.14 |  |
| Stress | 1.00 | 4.00 | 2.27 | .66 |  |
| Caffeine Use | .00 | 9.25 | 2.46 | 1.63 |  |
|  |  |  |  |  |  |

Supplemental Table 5: Daily Links between Sleep and Interview Variables

|  |  | Alertness | WASO | Sleep Time | Sleep  Quality | Stress | Self  Care | Utility | Rapport | Resistance | Difficulty |
| --- | --- | --- | --- | --- | --- | --- | --- | --- | --- | --- | --- |
| Alertness | rho |  |  |  |  |  |  |  |  |  |  |
|  | r | 1 |  |  |  |  |  |  |  |  |  |
| WASO | rho | -.28^**^ |  |  |  |  |  |  |  |  |  |
|  | r | -.27^**^ | 1 |  |  |  |  |  |  |  |  |
| Sleep Time | rho | .38^**^ | -.02 |  |  |  |  |  |  |  |  |
|  | r | .33^**^ | .01 | 1 |  |  |  |  |  |  |  |
| Sleep Quality | rho | .19^**^ | -.18^**^ | .35^**^ |  |  |  |  |  |  |  |
|  | r | .23^**^ | -.16^**^ | .35^**^ | 1 |  |  |  |  |  |  |
| Stress | rho | -.09^*^ | -.01 | -.14^**^ | -.39^**^ |  |  |  |  |  |  |
|  | r | -.14^**^ | .00 | -.15^**^ | -.39^**^ | 1 |  |  |  |  |  |
| Self Care | rho | -.03 | .07 | .07 | .21^**^ | -.29^**^ |  |  |  |  |  |
|  | r | -.02 | .11^**^ | .05 | .17^**^ | -.27^**^ | 1 |  |  |  |  |
| Utility | rho | .04 | .04 | -.12 | .13 | .07 | .20^**^ |  |  |  |  |
|  | r | .07 | .04 | -.12 | .13 | .06 | .14^*^ | 1 |  |  |  |
| Rapport | rho | .05 | .06 | -.08 | .11 | -.04 | .17^*^ | .52^**^ |  |  |  |
|  | r | .11 | .04 | -.11 | .12 | -.03 | .14 | .53^**^ | 1 |  |  |
| Resistance | rho | -.04 | -.05 | .00 | -.18^**^ | .17^*^ | -.12 | -.51^**^ | -.62^**^ |  |  |
|  | r | **-.18^*^** | -.04 | .04 | -.19^**^ | .17^*^ | -.15^*^ | -.51^**^ | -.66^**^ | 1 |  |
| Difficulty | rho | -.07 | -.13 | -.09 | -.30^**^ | .37^**^ | -.10 | -.23^**^ | -.26^**^ | .40^**^ |  |
|  | r | **-.18^*^** | -.01 | -.11 | -.34^**^ | .41^**^ | -.10 | -.17^*^ | -.23^**^ | .37^**^ | 1 |
